# From epidemiology to precision prevention: Why the latest breast cancer roadmap deserves your attention

**DOI:** 10.14440/jbm.0276

**Published:** 2025-10-07

**Authors:** Ralf Weiskirchen

**Affiliations:** Institute of Molecular Pathobiochemistry, Experimental Gene Therapy and Clinical Chemistry (IFMPEGKC), RWTH University Hospital Aachen, Aachen D-52074, Germany

This editorial discusses the recent review by Anton *et al.*,^1^ highlighting its comprehensive synthesis of breast cancer epidemiology, genetic susceptibility, lifestyle, and environmental risk factors, as well as state-of-the-art screening recommendations. By integrating high-penetrance germline mutations, emerging polygenic risk scores, and modifiable exposures into a unified framework, the review offers clinicians and researchers an actionable blueprint for personalized prevention and early detection. The commentary emphasizes the clinical significance, methodological robustness, and public health implications of this article, arguing that it is a must read for anyone engaged in the multidisciplinary management of breast cancer.

Breast cancer is a significant issue due to its high prevalence, personal impact, and global economic burden.^2^ It is the most common cancer in women and roughly accounts for one-third of all malignancies in women, with a mortality rate constituting about 15% of the total number of cases diagnosed.^3^ The disease can result in complex treatments, chronic side effects, and emotional challenges such as anxiety and depression. The rising incidence, especially in low- and middle-income areas, adds financial strain to healthcare systems, with costs reaching billions of dollars annually in high-income countries.^4^ The indirect costs of lost productivity and caregiver time further emphasize the societal and economic impact of breast cancer.

In a thorough review that combines epidemiology, genetics, lifestyle factors, and imaging to explore breast cancer susceptibility and prevention, Anton *et al*.^1^ highlight the increasing burden of breast cancer worldwide and provide a transparent overview of the topic from 2013 to 2025. The review takes a life-course approach to risk factors, separating those that cannot be changed, like age and genetics, from modifiable factors like alcohol consumption and smoking. They emphasize the impact of lifestyle choices on risk, such as the increased risk associated with alcohol intake. By considering different stages of life, from infancy to menopause, the authors suggest opportunities for intervention at both the public health and individual levels.

The genetic landscape relevant to breast cancer is equally discussed. High- and intermediate-penetrance genes such as BRCA1 DNA repair-associated protein (*BRCA1*, OMIM: 113705), BRCA2 DNA repair-associated protein (*BRCA2*, OMIM:600185), tumor protein p53 (TP53, OMIM:191170), partner and localizer of BRCA2 (*PALB2*, OMIM:610355), ATM serine/threonine kinase (*ATM*, OMIM: 607585), checkpoint kinase 2 (*CHEK2*, OMIM: 604373), RAD51 paralog c (*RAD51C*, OMIM: 602774), and Rad51 paralog D (*RAD51D*, OMIM: 602954) are discussed along with their lifetime-risk estimates and tumor-subtype correlations. It is emphasized that mutations in *BRCA1/2* alone account for only 5–10% of all cases. Importantly, the authors draw attention to more than 180 low-penetrance loci discovered by genome-wide association studies, which together explain roughly 18% of the missing heritability and suggest a future of polygenic risk modeling. The identification of novel peroxisomal candidate genes in families who test negative for known mutations underscores how much discovery remains ahead.

When discussing early detection, the review provides a well-rounded approach by offering practical advice while also evaluating different technologies. The authors discuss that digital mammography is still considered the most effective method with 99% specificity and 78% sensitivity.^1^ However, the authors demonstrate how tomosynthesis can improve the visibility of lesions in dense breasts and how magnetic resonance imaging (MRI) is more sensitive in high-risk women, detecting an additional 4.2 cancers/1,000 screenings. The review also includes clear recommendations based on age and risk factors. For example, it suggests starting annual MRI and mammography screenings between the ages of 25 and 30 for individuals with *BRCA* mutations, or ten years before the youngest affected relative for cases of familial breast cancer. These recommendations help clinicians translate scientific knowledge into practical algorithms for early detection.

By integrating lifestyle, environmental, hormonal, and genetic data into a unified framework, this review enhances the precision-prevention paradigm. This allows for more precise estimation of absolute risk and the development of personalized surveillance and chemoprevention strategies. The detailed tables, clear explanations of molecular mechanisms, and identification of research gaps will assist clinicians in need of immediate guidance, epidemiologists who plan to conduct cohort studies, and policymakers involved in formulating public health interventions.

In addition to summarizing established science, the authors effectively emphasize practical strategies for change. They quantified how minor adjustments in alcohol consumption, weight management, or night-shift scheduling could lead to significant reductions in disease incidence. This empowers clinicians to discuss prevention with their patients in tangible terms. From a clinical perspective, the review supports the use of multi-gene panel testing and provides detailed guidance on interpreting genetic variants. This will speed up the shift from focusing solely on detection via *BRCA* genes to a more comprehensive genomic risk assessment approach. As a result, individuals with *PALB2*, *CHEK2*, *RAD51C*, or *RAD51D* mutations will no longer be overlooked in screening programs. This is in agreement with previous reports proposing to add these genes into routine genetic testing for cancer risk prediction and to refine treatment algorithms.^5^-^7^

Finally, by highlighting disparities in incidence, mortality, and breastfeeding rates among different racial and socioeconomic groups, the review effectively argues for integrating genetic services, high-quality imaging, and lifestyle intervention resources into underserved communities. This approach aligns the next decade of breast cancer control with the principles of equity and precision medicine.

In summary, this meticulous, data-rich, and forward-looking synthesis combines rigorous methodology with clear, clinically actionable insights. Incorporating balanced insights on biology, behavior, and technology into their review, Anton *et al*.^1^ provide a groundbreaking roadmap that not only clarifies existing knowledge but also outlines a path toward customized, risk-adjusted care for individuals at risk of breast cancer, offering an indispensable primer for anyone dedicated to understanding, preventing, or managing breast cancer.
